# An Incidence of Duplicated Princeps Pollicis and Radialis Indicis Arteries

**DOI:** 10.7759/cureus.14894

**Published:** 2021-05-07

**Authors:** Nicholas Lampasona, Taylor Mazzei, Brandon LaPorte, Arthur Speziale, Oleg Tsvyetayev, Gary Schwartz, Nicholas Lutfi

**Affiliations:** 1 Osteopathic Medicine, Nova Southeastern University Dr. Kiran C. Patel College of Osteopathic Medicine, Fort Lauderdale, USA; 2 Orthopaedic Surgery, Nova Southeastern University Dr. Kiran C. Patel College of Osteopathic Medicine, Fort Lauderdale, USA; 3 Internal Medicine, Nova Southeastern University Dr. Kiran C. Patel College of Osteopathic Medicine, Fort Lauderdale, USA; 4 Radiology, Nova Southeastern University Dr. Kiran C. Patel College of Osteopathic Medicine, Fort Lauderdale, USA; 5 Orthopaedic Surgery, Nova Southeastern University Dr. Kiran C. Patel College of Allopathic Medicine, Fort Lauderdale, USA; 6 Anatomy, Nova Southeastern University Dr. Kiran C. Patel College of Osteopathic Medicine, Fort Lauderdale, USA

**Keywords:** princeps pollicis artery, anatomic anomaly, hand surgery, superficial palmar arch, deep palmar arch, superficial radial artery, deep radial artery, radialis indicis

## Abstract

The princeps pollicis artery (PPA) is typically a direct branch off the deep palmar arterial arch. Identified is a 90-year-old female cadaver in which the right hand has a duplicated PPA and radialis indicis (RI) artery. These vessels originate from the superficial palmar arterial arch as variant vessels as well as from the deep palmar arterial arch. The superficial arch appears in its classic pattern, while the duplicate PPA and RI present at the radial aspect of the superficial arch in the volar first web space with clear communication to the superficial radial artery. There are many common surgical procedures that require precise knowledge of the first web space, such as Dupuytren's contracture release, trigger thumb release, and syndactyly release at the first web space. Further, percutaneous pinning of fractures at the base of the thumb may pose an inherent risk to the underlying vessels. Understanding these variations of hand vasculature is of clinical significance in disciplines such as orthopedic surgery, plastic surgery and vascular surgery.

## Introduction

This case report has been presented as a poster at the 2020 Student Osteopathic Surgical Association Spring Convention in Des Moines Iowa on February 20, 2020, and the Consortium for Excellence in Medical Education in April 2020, online. Blood supply to the hand is a result of two anastomosing arches, which arise from the two main arteries of the forearm - the ulnar artery and radial artery. The ulnar artery produces the superficial palmar arterial arch, which is the primary branch responsible for supplying the palm and fingers. The deep palmar arterial arch is a terminal branch of the radial artery that is responsible for supplying the deep hand. The princeps pollicis artery (PPA) and radialis indicis (RI) are typically direct branches off the deep palmar arterial arch. The PPA has been used in reference to the first palmar metacarpal artery or simply the primary source of blood supply to the thumb [1].

The PPA is typically one of the terminal branches of the radial artery. It continues to travel distally along the ulnar surface of the first metacarpal and dorsal to the origin of the adductor pollicis muscle. As the artery traverses, it sends off branches that feed the proximal half of the first metacarpal. Upon reaching the neck of the first metacarpal, it then sends off two circumflex branches - the ulnar circumflex branch and radial circumflex branch. The ulnar circumflex branch runs inferior to the tendon of the adductor pollicis to anastomose with a branch of the dorsal metacarpal artery. The radial circumflex branch runs laterally between the insertions of the opponens pollicis and the flexor pollicis brevis muscles to anastomose with a branch of the dorsal arteries of the back of the metacarpal bones [1]. The PPA then bifurcates at the head of the first metacarpal into the ulnar terminal branch and the radial terminal branch. The ulnar terminal branch receives a branch of the first common palmar digital artery and then continues distally along the medial aspect of the phalanges as the ulnaris pollicis artery. The radial terminal branch receives a branch from the marginalis palmaris radialis artery and then continues distally along the phalanges as the radialis pollicis artery. The ulnaris pollicis artery and radialis pollicis artery has a proximal transverse anastomosis at the proximal phalanges [1].

It is not uncommon to have anatomical variations with blood supply to the hand, which has been noted in multiple studies [1-3]. According to Patnaik et al., aberrations in the superficial palmar arch are much more common than the deep palmar arch and these variations have been noted in multiple studies [2]. Understanding these variations is of clinical significance in disciplines such as orthopedic surgery, plastic surgery and vascular surgery. As the area of microvascular hand surgery continues to progress, precise knowledge is required to minimize adverse events from replantation, revascularization, or composite tissue transfer [4]. There are many common surgical procedures that require precise knowledge of the first web space where the PPA and RI can be encountered. These procedures include Dupuytren's contracture release, trigger finger release to the thumb, and syndactyly release at the first web space.

## Case presentation

**Figure 1:**
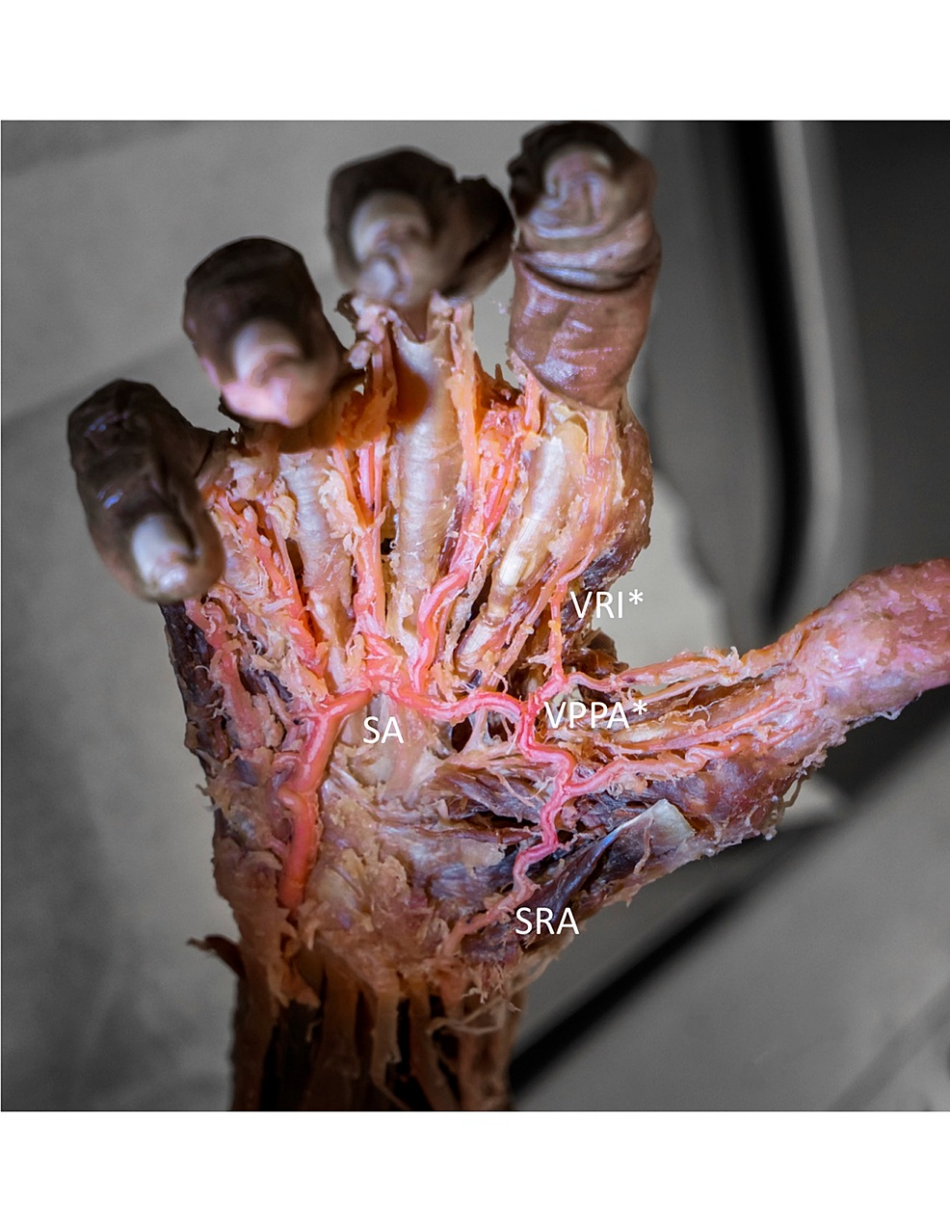
Right hand, anterior/posterior view illustrating the radialis indicis occurring from the SA illustrated as VRI, princeps pollicis artery occurring from the superficial palmar arch illustrated as VPPA and the SRA in clear communication with the SA. SA: Superficial palmar arch VRI: Volar radialis indicis VPPA: Volar princeps pollicis artery SRA: Superficial radial artery

**Figure 2:**
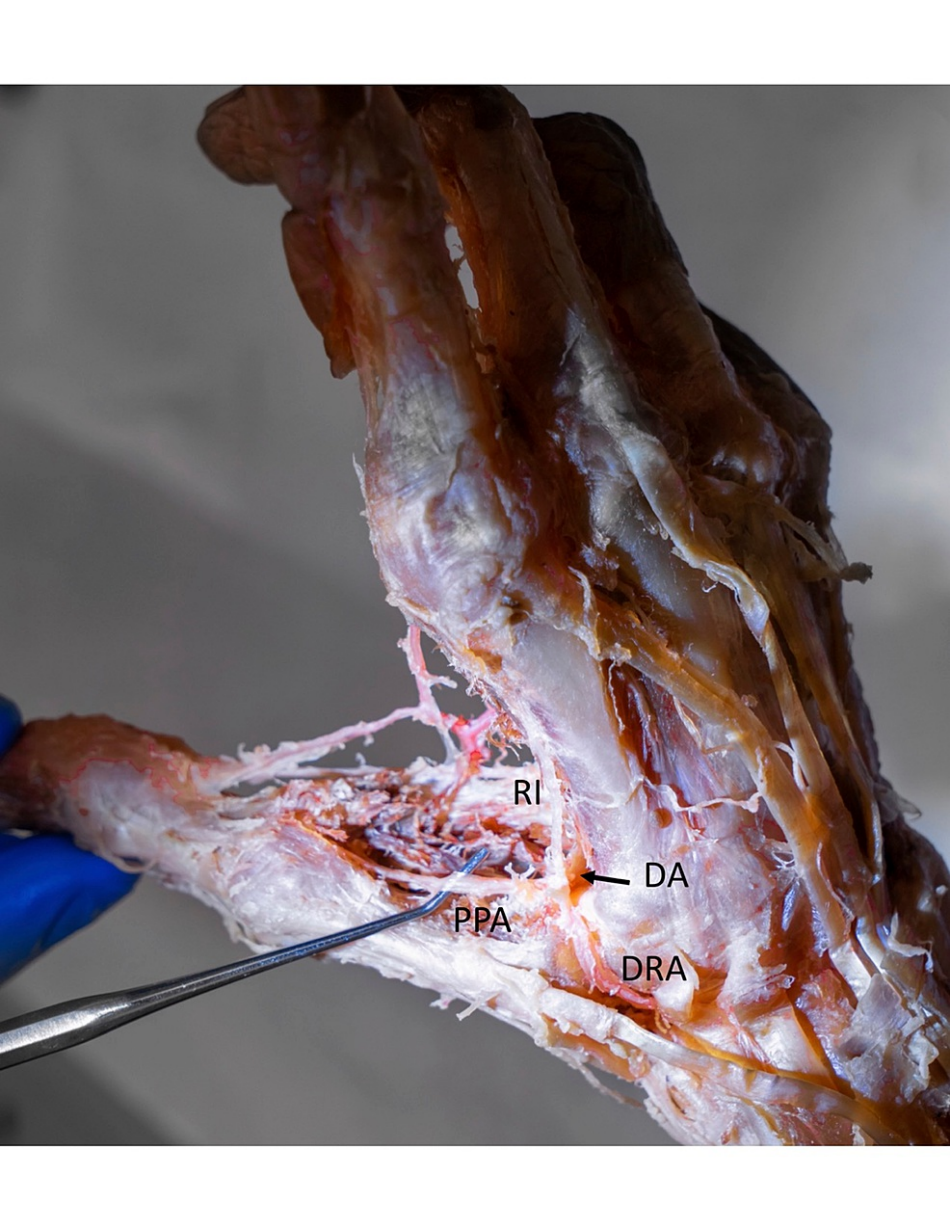
Right hand, oblique view illustrating the deep palmar arch as anatomically expected. The DRA gives rise to the RI, PPA, and DA. The DA travels directly in the ulnar direction from the origin of the RI and PPA at the level of the metacarpals towards the ulnar side of the hand. DRA: Deep radial artery RI: Radialis indicis PPA: Princeps pollicis artery DA: Deep palmar arch

Identified is a 90-year-old female, with no obvious deformity of the hands or bilateral upper extremities and no obvious surgical procedures performed on the hands or bilateral upper extremities. The right hand is shown to have a duplicate PPA as well as a duplicate RI emerging from the superficial palmar arch in communication with the superficial radial artery (Figure 1). This cadaver also has an intact PPA, as well as RI emerging from the deep palmar arch as anatomically expected (Figure 2).

An initial approach to the vasculature of the right hand was done volarly. After superficial dissection of the palmar fascia, the superficial palmar arch was visualized. In addition, the superficial radial artery was visualized at the radial aspect of the wrist directly communicating with the superficial arch. A small arterial branch, approximately 2 mm in length was visualized emerging from the superficial palmar arch in the volar first web space further bifurcating into a PPA and RI. This PPA continued along the ulnar aspect of the first metacarpal (Figure 1). The RI can be seen coursing distally along the radial aspect of the second metacarpal (Figure 1).

In an attempt to ascertain any contribution of blood supply to the thumb from the deep palmar arch, the deep branch of the radial artery was dissected at the anatomical snuffbox. The artery was followed distally into the dorsal first web space. At the level of the metacarpotrapezial joint, the deep radial artery gave off three branches: (1) the PPA, which courses along the ulnar side of the first metacarpal extending distally towards the phalanges of the thumb. (2) The deep palmar arch traveling in an ulnar direction across the volar hand at the level of the metacarpals. (3) The RI coursing along the radial aspect of the second metacarpal (Figure 2). Approximately 3 mm proximal to the interphalangeal joint of the thumb, the PPA further divides into the radial proper digital artery of the thumb and ulnar proper digital artery of the thumb. These PPA and RI vessels from the deep radial artery have no communication with the previously described PPA and RI found emerging from the superficial palmar arch. Thus, it has been determined that there is duplicate PPA and RI both superficially and deep in this cadaveric specimen.

## Discussion

Anatomical variations in the princeps pollicis have been described in previous studies. Adachi noted some variations in 1928. He classified the distal segment of the artery into two categories by its relation to the adductor pollicis muscle. The distal segment was categorized into type a or type b referenced to a volar or dorsal course to the tendon of the adductor pollicis muscle, respectively [3]. However, this classification is limited since the full disposition of the PPA is lacking; the variability in the artery’s origin and proximal course is neglected. Murakami et al. recognized this limitation and created a similar system where seven different classification types were used for the proximal segment of the PPA (type I-type VII) and two different classification types proposed by Adachi continued to be used for the distal segment of the PPA (type a and type b). Murakami et al. state that type Ia is classically considered normal since it is analogous to the first metacarpal artery, which was observed in 42 of 90 Japanese cadaveric specimens [1]. It is important to note that these variations are in reference to the PPA coming off the deep arterial arch, there was no reference to a duplicate PPA or a PPA coming off the superficial arterial arch since it was not observed in their study. A previous cadaveric case study reports an incidence of the PPA originating from the superficial palmar arch with no communication from the radial artery, however, neglected to illustrate or discuss the deep arterial arch, making it unclear whether or not there is any contribution of a PPA from the deep arch [5]. Further dissection and illustration in this case would have proved/disproved any alternate blood supply to the thumb [5].

Clinical importance of a duplicate PPA can be shown through symptom presentation and subsequent complications and sequelae. Fracture repairs at the base of the first metacarpal, such as in the case of a Bennett fracture, may require percutaneous pinning using Kirschner wire (K-wires) [6]. If a clinician is not aware of possible vascular variation in the first web space during closed reduction and percutaneous K-wire pinning, damage to underlying vasculature is possible. Knowledge of the blood supply to the thumb and possible variations may provide an insight into symptom management, diagnosis and clinical treatment without complication and sequelae.

Treatment such as thenar flap surgery and thumb amputations involving replantation may be affected by vascular anomalies of the hand as found here. In the case of a duplicate PPA, one must consider more blood flow to a thenar flap through the duplicate artery. The proposed time to keep a thenar flap intact is two weeks [7]. This time could theoretically be shortened with increased blood flow due to a duplicate blood supply, as found here. This decreased time could reduce the risk of postoperative complications.

In the incidence of total thumb amputation, great toe transfers are the treatment of choice for plastic surgery; however, these transfers have been reviewed as complex [8]. One complexity of this surgical procedure is the potential complication of vascular spasm. A review of 300 free toe transfers also finds that the caliber of vessel diameter may influence vascular spasm [9]. In which case, the option of a duplicate vessel as found here may pose an advantage to a surgeon to match vessel diameters of the transferred digit.

Cadaveric dissection has been a common practice by medical students for centuries. In contemporary times, medical student preparation prior to dissection may include reading dissection manuals, watching online dissection videos, and using medical atlases. Typically, dissection laboratory manuals and basic human anatomy atlases do not show branches from the superficial palmar arch to the thumb. The volar side of the hand has many superficial structures that must be carefully dissected, ergo without knowledge of this PPA variation it could potentially be dissected out in the process, therefore, potentially contributing to why this anatomical variation may be under reported in literature. More research should be done with special attention to the superficial radial artery to see the incidence of this specific variation. As technology advances, more medical schools are moving away from traditional cadaver labs and towards virtual platform. This may reduce students’ tactile skill as well as their observation of anatomy in a “non-textbook” manner.

## Conclusions

This case report explores an incidence of duplicated PPA and RI occurring from the superficial palmar arch. An intact PPA and RI artery are described as anatomically expected from the deep palmar arch. To the best of the authors’ knowledge, this anatomic anomaly has not been fully described in medical literature except for in one other case report, which lacks description of the full vasculature to the thumb. Much of the discussion surrounds the clinical implications of such anomaly. Further research on cadavers with special attention to the volar first web space may help establish an increase prevalence of vascular variations of the superficial and deep palmar arches.
